# Transcriptomic profiles of 33 opium poppy samples in different tissues, growth phases, and cultivars

**DOI:** 10.1038/s41597-019-0082-x

**Published:** 2019-05-20

**Authors:** Yucheng Zhao, Zhaoping Zhang, Mingzhi Li, Jun Luo, Fang Chen, Yongfu Gong, Yanrong Li, Yujie Wei, Yujie Su, Lingyi Kong

**Affiliations:** 1 Jiangsu Key Laboratory of Bioactive Natural Product Research and State Key Laboratory of Natural Medicines, School of Traditional Chinese Pharmacy, China Pharmaceutical University, No. 24 Tongjiaxiang, Nanjing, 210009 China; 2 China Agriculture Research System (CARS-21), No. 234 Xinzhen Road, Huangyang town, Liangzhou District, Wuwei, Gansu 733006 China; 3 Genepioneer Biotechnologies Co. Ltd., No. 9 Weidi Road, Qixia District, Nanjing, 210014 China

**Keywords:** Secondary metabolism, Plant genetics

## Abstract

Opium poppy is one of the most important medicinal plants and remains the only commercial resource of morphinan-based painkillers. However, little is known about the regulatory mechanisms involved in benzylisoquinoline alkaloids (BIAs) biosynthesis in opium poppy. Herein, the full-length transcriptome dataset of opium poppy was constructed for the first time in accompanied with the 33 samples of Illumina transcriptome data from different tissues, growth phases and cultivars. The long-read sequencing produced 902,140 raw reads with 55,114 high-quality transcripts, and short-read sequencing produced 1,923,679,864 clean reads with an average Q30 rate of 93%. The high-quality transcripts were subsequently quantified using the short reads, and the expression of each unigene among different samples was calculated as reads per kilobase per million mapped reads (RPKM). These data provide a foundation for opium poppy transcriptomic analysis, which may aid in capturing splice variants and some non-coding RNAs involved in the regulation of BIAs biosynthesis. It can also be used for genome assembly and annotation which will favor in new transcript identification.

## Abstract (summary)

**Table:** 

Design Type(s)	transcription profiling design • sequence annotation objective • organism part comparison design
Measurement Type(s)	transcription profiling assay
Technology Type(s)	RNA sequencing
Factor Type(s)	cultivar • developmental stage • organism part
Sample Characteristic(s)	Papaver somniferum • root • stem • leaf

Machine-accessible metadata file describing the reported data (ISA-Tab format)

## Background & Summary

Opium poppy (*Papaver somniferum*) is one of the most important medicinal plants in the world and remains the only commercial resource of morphinan-based painkillers^1^. Its main active ingredient, BIAs, also displays potential pharmacological activity in relieving cough, muscle relaxation, anticancer, and so on^2,3^. Although approximately 100,000 hectares (ha) of opium poppy are cultivated annually worldwide, this insufficient to meet the demand for managing moderate or severe pain^1,4^. Engineered microbes could be used to produce BIAs such as opiates and noscapine^5–7^, however, major hurdles, such as low yield and unclear biosynthetic pathways, make it different to scale-up this method of production for most BIAs. Hence, how to guarantee the source of BIAs to meet the medical applications, such as in pain relief or palliative care, has been recognized as a major issue that needs to be resolved.

Investigation into the BIA biosynthesis mechanism began in the 1960s with radiotracer technology^3^. The emergence of transcriptomics, proteomics, and metabolomics, coupled with recent genome analysis tools, accelerated the discovery of new BIA biosynthetic genes that could facilitate metabolic engineering reconstitution of commercial source of valuable BIAs in microbes^1,5,7–11^. However, some key steps in the BIA biosynthesis pathway are yet to be identified, and there has been a lack of investigation into the molecular mechanisms of gene regulation in the pathway^3^. In addition, there are few reports major in the processes of BIAs biosynthesis, such as compound dynamic accumulation, tissue-specific distribution, enzyme interactions and metabolism, compartmentalization and transport^3^. Therefore, investigation into the regulation mechanism and understanding the compound accumulation process is a key way to improve the yield of BIAs.

Of all the strategies in metabolic regulation, over-expression and silencing of genes involved in the metabolism of target compounds are the most widely used method. However, non-coding RNAs, alternative splicing/translation/polyadenylation (AS/AT/APA), formation of heterodimers, and gene fusion have also been shown to increase the flexibility of the transcriptome, functional complexity of plants, and the trend of metabolic flow, tissue-specific accumulation or product yield^8,9,12,13^. However, little research has been conducted on the regulatory mechanisms in opium poppy despite the recently release of its genome^1^. The reason for this oversight may be the lack of the transcriptional information on opium poppy in different growth periods/status or tissues. In addition, the short sequences created by third-generation sequencing and the gene information in DNA standard could not capture the AS/AT/APA of transcripts^1,3^.

**Fig. 1:**
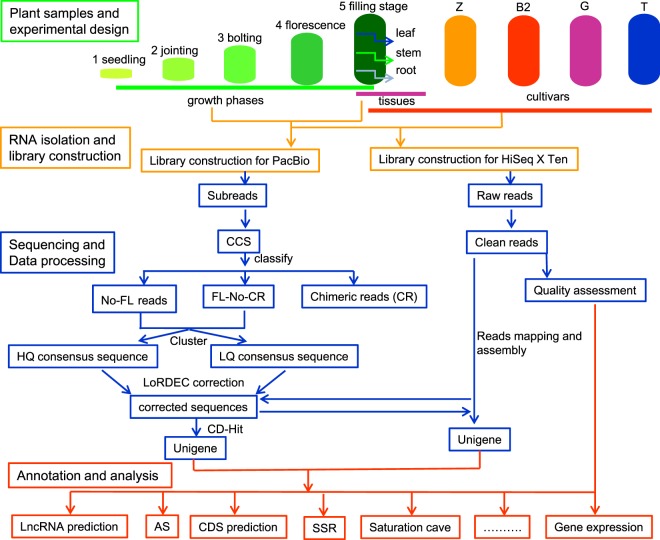
Overview of the experimental design and the data processing pipeline. This study consisted of experimental design, RNA isolation, sequencing and data processing, annotation, and analysis, all of which are marked with different colors. Samples were divided into different growth phases, tissues, and cultivars. The numbers 1–5 indicate the five growth phases of opium poppy. Samples in the filling stage were used for either growth phases or tissues and cultivars. All 33 samples were subject to Illumina sequencing, and only samples from B1 were used for PacBio Sequel sequencing.

**Fig. 2:**
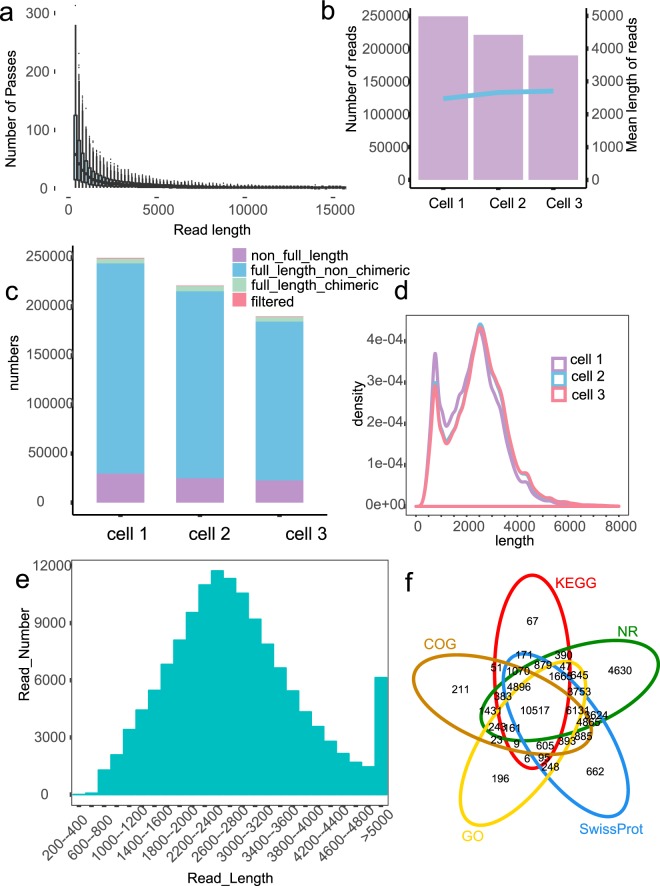
Output and quality assessment of the SMRT data. (**a**) Passes vs. read length. (**b**) Read length of inserts in three cells. (**c**) Read number of each type of read in three cells. (**d**) Density of full-length non-chimeric reads in three cells. (**e**) Length and number of mRNA without redundancy. (**f**) Venn graph of annotation.

**Fig. 3:**
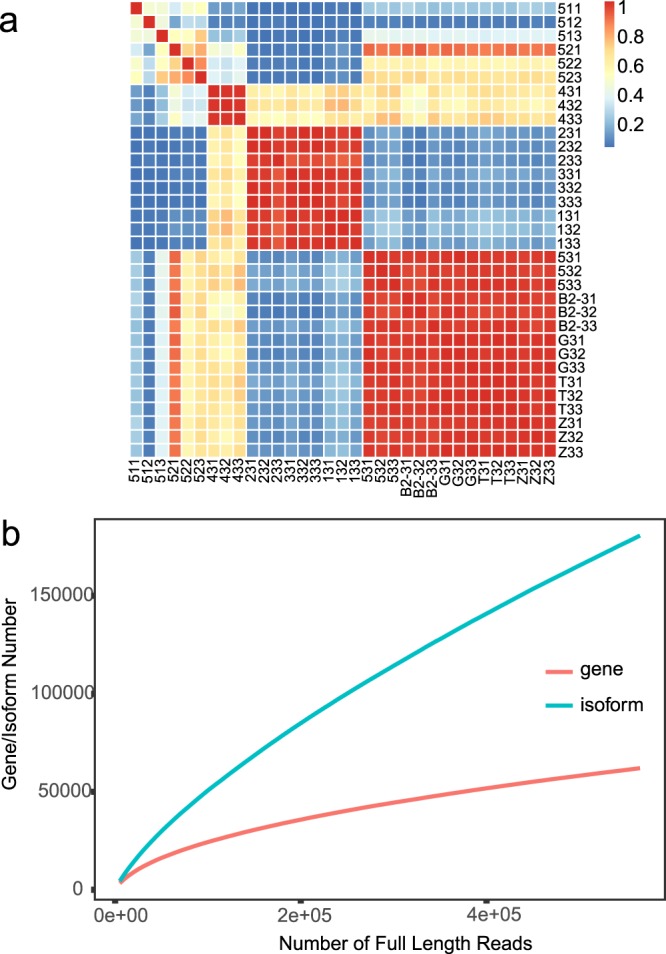
Correlation analysis of repeated samples and saturation curve of transcripts and genes. (**a**) Correlation analysis of repeated samples. Heatmap displaying the similarities among all samples based on Poisson distances. (**b**) Saturation curve of transcripts and genes. The saturation curve shows with the incense of FL reads, the number of genes tends to remain flat, while the number of transcripts rises rapidly.

**Fig. 4:**
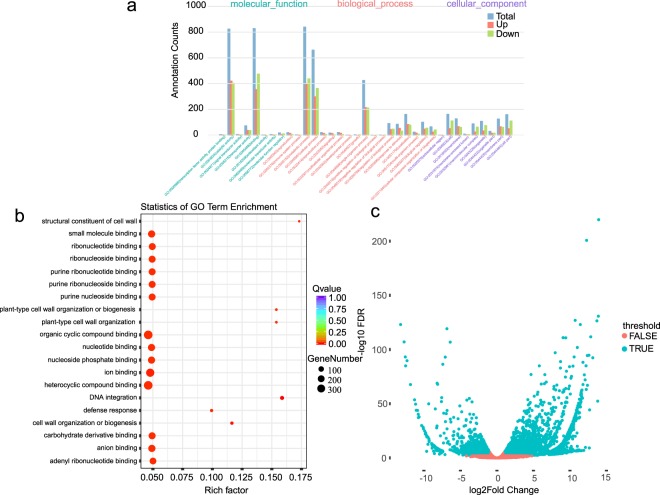
Example of differential gene expression analysis and the enrichment in GO between B2 and ZB. (**a**) GO classification map of differential expression genes. (**b**) Statistics of GO enrichment. (**c**) Volcano map of differential expression genes.

Third-generation single-molecule real-time (SMRT, Pacific Biosciences) sequencing has increasingly been used to detect AS/AT/APA, to identify novel isoforms, to predict non-coding RNA, and for gene fusion studies due to its long reads^14,15^. However, to date, there has been no report on a full-length transcriptome dataset of opium poppy, despite the recent release of its genome^1^. Herein, we construct the full-length transcriptome dataset of opium poppy using twenty-one pooled RNA samples from three different tissues and five different growth phases (Fig. 1). This produced 902,140 post-filter polymerase reads and 660,418 circular consensus sequence (CCS) reads (Table 1 and Fig. 2). After data processing, 566,746 full-length (FL) reads, 180,511 non-redundant isoforms and 61,856 unigenes (59,144 protein-coding unigenes and 2,712 non-coding unigenes) were obtained for functional annotation (Fig. 2). In addition, a total of 1,923,679,864 clean, paired-end short reads were produced (Fig. 3 and Table 2). Gene expression levels were then determined using RSEM and converted into fragments per kb per million fragments (FPKM) value (Fig. 4)^16,17^. The dataset reported here, provides an overview of the gene expression levels, AS/AT/APA, and full-length transcript/unigene/mRNA of the opium poppy during key statuses. It can also be used to analyze the regulation mechanisms of BIAs biosynthesis according to its tissue-specific distribution, dynamic accumulation in different growth phases as well as BIAs diversity in different germplasm resources.

**Table 1:** Summary of post-filter polymerase reads of long-read sequencing.

Cell Name	Polymerase Bases	Polymerase reads	Mean polymerase reads length	N50	Mean insert length	Subreads Bases	Subreads	Mean subreads length	Subreads N50
Cell 1	7,134,964,542	290,143	24,591	42,250	3,192	6,896,883,917	3,684,400	1,871.92	2,604
Cell 2	5,637,070,764	271,411	20,770	38,750	3,047	5,481,138,738	2,865,188	1,913.01	2,663
Cell 3	6,993,388,572	340,586	20,533	36,250	3,036	6,700,617,834	3,825,257	1,751.68	2,450

**Table 2:** Statistics of Illumina-based RNA-seq data and quantification of gene expression.

Sample	Index	Clean Bases	Q30 Rate (%)	RPKM			
				0–0.1	0.1–3.75	3.75–15	>15
131	TTAGGC	8713642719	95	16,032	29,642	9,636	6,546
132	TGACCA	9564232510	96	16,532	29,732	9,285	6,307
133	ACAGTG	8107787693	96	16,599	29,566	9,314	6,377
231	GCCAAT	9856735582	96	14,703	28,829	11,103	7,221
231	CAGATC	9135096761	96	14,664	28,591	11,303	7,298
233	ACTTGA	8796177410	96	14,738	27,346	11,713	8,059
331	GATCAG	9098543425	96	16,710	29,501	9,491	6,154
332	TAGCTT	10710694418	96	15,936	29,443	9,956	6,521
333	GGCTAC	9433383049	96	15,573	29,564	10,177	6,542
431	CTTGTA	8512385396	96	15,180	28,368	10,846	7,462
432	AGTCAA	8863916114	96	15,682	27,710	10,627	7,837
433	AGTTCC	9205308102	96	14,991	27,757	11,208	7,900
511	TGACCA	7080192660	94	15,480	26,114	12,024	8,238
512	ACAGTG	6668054022	96	15,392	26,004	11,811	8,649
513	GCCAAT	8814911815	96	15,470	28,361	10,500	7,525
521	CAGATC	8857730898	96	15,831	26,107	11,330	8,588
522	ACTTGA	8354907591	96	16,108	26,313	10,792	8,643
523	GATCAG	6919453352	96	17,249	28,572	8,458	7,577
531	TAGCTT	8282182945	96	15,611	28,124	10,408	7,713
532	GGCTAC	6896148722	95	15,771	28,693	9,966	7,426
533	CTTGTA	8631197062	95	14,860	28,393	10,683	7,920
B2-31	AGTCAA	8664717417	88	15,916	27,833	10,396	7,711
B2-32	AGTTCC	9101628243	90	16,114	27,576	10,248	7,918
B2-33	ATGTCA	7740678568	86	15,292	28,381	10,548	7,635
T31	CCGTCC	6488485876	95	16,674	27,689	10,025	7,468
T32	GTCCGC	8853003315	89	16,820	27,750	9,858	7,428
T33	GTGAAA	8213957582	90	16,707	27,532	10,081	7,536
Z31	GTGGCC	9127832844	89	16,843	27,659	9,898	7,456
Z32	GTTTCG	9417530235	87	16,940	28,042	9,500	7,374
Z33	CGTACG	9140750089	88	16,660	27,983	9,770	7,443
G31	GAGTGG	9615912781	89	16,337	27,959	10,022	7,538
G32	ACTGAT	9595710378	90	16,519	27,960	9,823	7,554
G33	ATTCCT	9773818624	90	16,086	28,398	9,997	7,375

## Methods

### Plant material and experimental design

All the opium poppy for this study was cultured in our experimental plot. The original cultivar was named B1 and represents the original state of opium poppy, known as wild opium poppy. Other germplasm resources resulting from directed breeding in our institute were named B2, T, G and Z. Their characteristics and major BIAs content are summarized in Tables 3 and 4, respectively. Samples were divided into different tissues, cultivars, and growth phases (Fig. 1). Samples from different tissues in B1 (root, stem, and leaves) and germplasm resources (B1, B2, T, G, ZB) were collected during filling stage on Jul 4, 2017. Samples in different growth phases (seedling, jointing, bolting, florescence, and filling) were collected from March to July of 2017. For each sampling point (eleven in all), three independent samples were collected. Unless otherwise mentioned, samples collected from opium poppy are all leaves. This produced 33 samples in all and their definitions of sampling are also listed in Table 3. The leaves from B1 at the filling stage were used as a shared sample for subsequent analysis: they were used as the filling stage sample when studying the growth rhythm of opium poppy, and also as a leaf sample in different tissues or a B1 sample in different cultivars. After washing and cleaning, the samples were immediately inundated with liquid nitrogen for 10 minutes and then stored in −80 °C freezers until use.

**Table 3:** Description and definition of the main characteristics of opium poppy in each germplasm lines, growth phase, and tissue.

Groups	Sample	Description
Germplasm lines	B1	Four white petals; white seed; plant height up to 115 cm and have an average leaves of 15; white flower with only one fruit
	B2	Two green sepals with 4–6 white petals; multi-branching; plant height up to 106 cm and usually have 13 leaves; white seed
	T	It has an average plant height of 110 cm and 12 leaves; White flower with purple spots; juice in white and filament in white; multi-branching which could produce at least three fruit
	Z	It has an average plant height of 105 cm and 11 leaves; White flower with purple spots; juice in light red and filament in white; multi-branching
	G	It has an highest plant height of 125 cm; 15 leaves; pink flower and golden anther; grey seed; juice in light red and filament in white
Growth phases	Seedling	Twenty days after sprouting
	Jointing	Sixty days after sprouting. In this time, a height of 50 cm stem could be observed
	Bolting	Seventy days after sprouting. In this time, a small bud was first observed at the tips of the stem
	Florescence	The date of opium poppy first bloom
	Filling stag	Fourteen days after falling flowers
Tissues	Root	The root in underground 5–8 cm part
	Stem	The middle position of the stem with a length of 3 cm
	Leaf	The top leaf of opium poppy

**Table 4:** The major BIAs contents in one Kilogram (mg/kg) dry leaves in different germplasm lines at filling stag.

BIAs name	B1	B2	G	T	Z
Morphine	599.9 ± 139.77	273.8 ± 17.68	700.0 ± 193.71	92.4 ± 14.64	807.8 ± 18.93
Codeine	44.7 ± 5.85	50.5 ± 34.67	266.7 ± 97.39	—	197.8 ± 79.43
Norcoclaurine	46.2 ± 13.25	14.9 ± 7.75	101.6 ± 55.20	55.1 ± 9.56	153.8 ± 89.71
Thebaine	—	—	—	16009.2 ± 605.32	—
Scoulerine	632.3 ± 116.43	629.0 ± 183.91	408.0 ± 5.54	107.8 ± 4.53	886.4 ± 222.46
Noscapine	58.3 ± 13.80	—	2021.9 ± 480.39	—	—
Papaverine	175.2 ± 44.88	118.9 ± 59.13	—	99.9 ± 12.25	—
Canadine	1150.9 ± 257.82	957.9 ± 203.02	1463. 3 ± 365.45	1641.6 ± 372.12	1021.3 ± 164.90
Sanguinarine	507.8 ± 23.82	417.4 ± 17.56	405.8 ± 9.41	—	—

— Is represented as the content of BIAs could not be detected. All data are represented as mean ± SD from three independent plants (n = 3). The unit is mg/kg dry leaves.

### RNA extraction, library preparation, and sequencing

For each sample, TRIzol Reagent (Tiangen Biotech Co., Ltd., Beijing, China) was used to extract RNA following the protocol provided by the manufacturer. After cDNA synthesis, samples were subjected to phosphorylation, “A” base addition, and end-repair according to library construction protocol. Sequencing adapters were then added to both sizes of the cDNA fragments. After PCR amplification of cDNA fragments, the 150–250 bp targets were cleaned up. We then performed paired-end sequencing on an Illumina HiSeq X Ten platform (Illumina Inc, CA, USA) (Fig. 1). For PacBio Sequel sequencing, RNA from 21 samples of B1 was mixed in equal amounts for reverse transcription using the Clontech SMARTer PCR cDNA Synthesis Kit (TaKaRa, Dalian, China). In order to determine the optimal amplification cycle number for the downstream large-scale PCR reactions, PCR cycle optimization was employed (PrimeSTAR® GXL DNA polymerase). Then the optimized cycle number was used to generate double-stranded cDNA. Large-scale PCR was performed for SMRTbell library construction (Pacific Biosciences). This include DNA damage repair, end repair, ligating sequencing adapters and removing fragments that failed to connect. Finally, the SMRTbell template was annealed to the sequencing primer, bound to polymerase, and sequenced on the PacBio Sequel platform using V2.1 chemistry (Pacific Biosciences) with 10-hour movies (Fig. 1).

### Data filtering, processing and yield

After sequencing, the raw reads were classified and clustered into a transcript consensus using the SMRT Link 5.1 pipeline (http://www.pacb.com/products-and-services/analytical-software/smrt-analysis/). After adaptor removal and elimination of low quality regions, we obtain 902,140 post-filter polymerase reads (19.77 GB) with an average length of 22 kb (Fig. 2 and Table 1). In order to improve the accuracy of sequencing, CCS reads were extracted from the subreads BAM file, which produced a total of 660,418 CCS reads with an average insert length of 2.61 kb (Fig. 2a,b). Briefly, CCS reads were extracted out of subreads.bam file with minimum full pass of 1 and a minimum read score of 0.8. CCS reads were then classified into full-length (FL) non-chimeric (NC), non-full-length (NFL), chimeras (C), and short reads based on cDNA primers and poly-A tail signal. Reads shorter than 50 bp were discarded. CCS reads with 5′ primer, 3′ primer and polyA tails were identified as FL reads, and 566,746 FL sequences ranging from 300 bp to 25,247 bp were obtained (Fig. 2c,d). Subsequently, the full-length non-chimeric (FLNC) reads were clustered by Iterative Clustering for Error Correction (ICE) software to generate the cluster consensus isoforms^18^. NFL reads were used by Arrow software to polish the obtained cluster consensus isoforms to obtain the final 55,114 FL polished high quality consensus sequences (accuracy ≥ 99%)^18^. Lordec was used to correct FL transcripts and CD-HIT was used to remove redundant sequences according to sequence similarity of high-quality transcripts^19,20^. Finally, 180,511 non-redundant isoforms and 61,856 unigenes were obtained for functional annotation (Fig. 2e,f). For Illumina paired-end RNA-seq, the low-quality reads (reads containing sequencing adaptors, reads containing sequencing primers, nucleotide with q quality score lower than 20) were removed. After that, a total of 1,923,679,864 clean, paired-end reads were produced (Table 2 and Fig. 3a). Illumina clean data was mapped onto our SMRT sequencing data using hisat2 v2.05^21^. RSEM was used to identify gene expression levels, which were then converted into an FPKM value^16,17^. DESeq R package was used to analyze differential expression^22^. Fold change ≥2 and adjusted *P*-value < 0.05 were set as threshold for significance of gene expression differences between the two samples (Fig. 4).

### Major BIAs content measurement

To analysis the major BIAs content, some 0.5 g dry leaves in each sample of different germplasm lines at filling stag were employed according to our previous publicized method^23^. For details, the sample was sequentially extracted three times in methanol, with the help of ultrasonication, for 30 min at room temperature. Then, methanol extracts were combined and concentrated under reduced pressure conditions to a volume of 2 mL. At last, 10 μL concentrated extracts was subject to analysis using HPLC HPLC equipped with a reversed phase C18 column (XDB-C18, 5 mm; Agilent, USA) according to our publicized method. The BIAs content was show as weight (mg) in one Kilogram dry leaves (mg/kg) and listed in Table 4, and its raw data of individual measurements are available at Figshare^24^.

### Functional annotation

Functional annotations of the novel genes were performed using BLAST searching against public databases such as Swiss-Prot, GO (Gene Ontology), and KEGG (Kyoto Encyclopedia of Genes and Genomics)^25,26^.

## Data Records

The Illumina HiSeq X Ten data (different growth phases, cultivars and tissues) and PacBio Sequel sequencing data have been submitted to the Sequence Read Archive (SRA) of NCBI under accession numbers SRP173551^27^, SRP173565^28^, SRP173546^29^, and SRP173728^30^, respectively (Table 5). The functional annotation and gene expression (RPKM) information of high-quality transcripts and unigenes are deposited in Figshare and Gene Expression Omnibus (GEO) in NCBI^24,31^. The differential gene expression data among different samples was also deposited in Figshare^24^.

**Table 5:** Metadata and description of each of the 33 samples that were sequenced.

Groups	Study	Biosample	Sample title	Accession	Description
Growth phases	SRP173551	SAMN10600731	131	SRR8325944	Seedling leaf from B1
			132	SRR8325943	Seedling leaf from B1
			133	SRR8325942	Seedling leaf from B1
			231	SRR8325941	Jointing leaf from B1
			231	SRR8325940	Jointing leaf from B1
			233	SRR8325939	Jointing leaf from B1
			331	SRR8325938	Bolting leaf from B1
			332	SRR8325937	Bolting leaf from B1
			333	SRR8325946	Bolting leaf from B1
			431	SRR8325945	Florescence leaf from B1
			432	SRR8325936	Florescence leaf from B1
			433	SRR8325935	Florescence leaf from B1
Tissues	SRP173546	SAMN10600614	511	SRR8325831	Filling stag root from B1
			512	SRR8325832	Filling stag root from B1
			513	SRR8325829	Filling stag root from B1
			521	SRR8325830	Filling stag stem from B1
			522	SRR8325827	Filling stag stem from B1
			523	SRR8325828	Filling stag stem from B1
			531	SRR8325825	Filling stag leaf from B1
			532	SRR8325826	Filling stag leaf from B1
			533	SRR8325833	Filling stag leaf from B1
Cultivars	SRP173565	SAMN10601491	B2-31	SRR8327183	Filling stag leaf from B2
			B2-32	SRR8327182	Filling stag leaf from B2
			B2-33	SRR8327181	Filling stag leaf from B2
		SAMN11104145	T31	SRR8327180	Filling stag leaf from T
			T32	SRR8327187	Filling stag leaf from T
			T33	SRR8327186	Filling stag leaf from T
		SAMN11104146	Z31	SRR8327185	Filling stag leaf from Z
			Z32	SRR8327184	Filling stag leaf from Z
			Z33	SRR8327178	Filling stag leaf from Z
		SAMN11104144	G31	SRR8327177	Filling stag leaf from G
			G32	SRR8327176	Filling stag leaf from G
			G33	SRR8327179	Filling stag leaf from G

## Technical Validation

### RNA quality control

The integrity of RNA sample was determined using the Agilent 2100 Bioanalyzer (Agilent Technologies, USA) and agarose gel electrophoresis. The purity and concentration of RNA samples were determined with the Nanodrop microspectrophotometer (Thermo Fisher Scientific, USA). For Illumina sequencing, 33 high-quality RNA samples (OD260/280 = 1.7~2.3, OD260/230 ≥ 2.0, RIN ≥ 7) were used to construct the sequencing library. The OD260/280, OD260/230, and RIN values for all RNA samples are listed in Table 6.

**Table 6:** RNA sample quality used in this study.

Sample	260/280	260/230	RIN	28 s/18 s
131	2.20	2.45	7.8	1.5
132	2.20	2.45	7.9	1.5
133	2.16	2.40	8.0	1.5
231	2.18	2.44	8.7	1.2
231	2.19	2.42	8.7	1.4
233	2.17	2.40	9.4	1.5
331	2.18	2.41	7.8	1.4
332	2.18	2.39	8.4	1.5
333	2.19	1.80	7.3	1.3
431	2.18	2.22	7.5	1.4
432	2.19	2.19	6.9	1.4
433	2.18	2.02	7.2	1.5
511	2.16	2.41	10	2.0
512	2.14	2.13	10	2.1
513	2.12	2.08	10	2.4
521	2.13	2.26	9.9	1.9
522	2.12	1.61	10	3.0
523	2.12	2.35	9.8	2.0
531	2.18	2.23	8.4	1.7
532	2.19	2.17	8	1.6
533	2.21	2.12	7.3	1.4
B2-31	2.18	2.45	7.2	1.4
B2-32	2.17	2.33	7.7	1.5
B2-33	2.18	2.35	7	1.3
T31	2.19	2.48	7.9	1.6
T32	2.18	2.46	7.4	1.5
T33	2.19	2.44	8	1.5
Z31	2.19	2.40	7.9	1.5
Z32	2.18	1.79	7.5	1.6
Z33	2.19	2.47	7.4	1.5
G31	2.17	2.40	8	1.7
G32	2.14	2.35	8.1	1.8
G33	2.14	2.45	7.9	1.7

### Quality evaluation of raw data

A high quality region finder was used to identify the longest region of a singly-loaded enzyme using a signal-to-noise ratio of 0.8 to filter out low-quality areas. In order to improve the accuracy of sequencing, the same polymerase reads were read multiple times in a closed loop, and the random error correction was then performed on the sequence read multiple times by the same insert fragment. This produced 660,418 CCS reads with a mean of 12.62 passes per read (Fig. 2a,b). FL reads were classified based on the location of and relationships between the 5′ primer, 3′ primer and polyA tail (Fig. 2c,d).

### Assessment of sample composition

For Illumina paired-end RNA-seq data, we measured the correlation coefficient and quantitative saturation of gene expression among 33 samples (biological repetition and biological variation, Fig. 3). Correlation of expression levels correlation among samples is an important index to test the reliability of experiments and the rationality of sample selection. Correlation coefficients close to 1 (red) indicate that the samples have a high similarity of expression patterns. If there is biological repetition in the sample, the correlation coefficient of biological repetition is usually higher. By calculating the relationship between the number of different full-length transcripts and the number of genes, we can observe whether the total number of genes has been measured up to the saturation level (Fig. 3b).

## ISA-Tab metadata file

Download metadata file

## Data Availability

SMRT Link 5.1 pipeline: http://www.pacb.com/products-and-services/analytical-software/smrt-analysis/. CD-HIT: http://www.bioinformatics.org/cd-hit/ (version 4.6.6). Blast: ftp://ftp.ncbi.nlm.nih.gov/blast/executables/blast+/LATEST/ (version 2.2.31).
